# Associations of Dyslipidemia with Dietary Intakes, Body Weight Status and Sociodemographic Factors among Adults in the United Arab Emirates

**DOI:** 10.3390/nu14163405

**Published:** 2022-08-19

**Authors:** Habiba I. Ali, Fadima Elmi, Lily Stojanovska, Naser Ibrahim, Leila Cheikh Ismail, Ayesha S. Al Dhaheri

**Affiliations:** 1 Department of Nutrition and Health, College of Medicine and Health Sciences, United Arab Emirates University, Al Ain P.O. Box 15551, United Arab Emirates; 2 Independent Researcher, Al Ain P.O. Box 67258, United Arab Emirates; 3 Institute of Health and Sport, Victoria University, P.O. Box 14428, Melbourne, VIC 8001, Australia; 4 Department of Clinical Nutrition and Dietetics, College of Health Sciences, University of Sharjah, Sharjah P.O. Box 27272, United Arab Emirates; 5 Nuffield Department of Women’s & Reproductive Health, University of Oxford, Oxford OX1 2JD, UK

**Keywords:** dyslipidemia, dietary intakes, Mediterranean diet, food frequency questionnaire, United Arab Emirates

## Abstract

Research on dietary and other factors associated with dyslipidemia in the United Arab Emirates (UAE) is limited. This study assessed the association of diet, body weight and other risk factors of dyslipidemia by conducting a cross-sectional survey among adults residing in three emirates of the UAE. Validated quantitative food frequency questionnaire and the WHO STEPS Instrument were used to assess dietary intakes, body weight and dyslipidemia-related diagnosis. Composite Mediterranean Diet Score was used to assess adherence to the Mediterranean Diet (MD). Of the 610 participants, dyslipidemia was reported by 23.5% of the 319 participants who ever had blood cholesterol levels measured. Self-reported dyslipidemia was associated with increased age, higher BMI, diabetes, hypertension and cardiovascular disease. Most participants did not meet the recommendations for dietary fiber and calorie intake from saturated fats (61.3% and 81.2%, respectively). Participants with dyslipidemia reported a higher median daily intake of vegetables compared to those without dyslipidemia (*p* < 0.001), who also showed a significantly higher intake of refined grains and sugar-sweetened beverages (*p* = 0.008). Participants aged ≥50 years were more likely to have adhered to the MD compared to 18–30-year old participants (OR = 4.16; 95% CI 2.59–6.69). Non-Emiratis had higher odds of adherence to the MD compared to UAE nationals (OR = 1.46; 95%CI 1.04–2.06). Interventions targeting behavioral risk factors of dyslipidemia are warranted.

## 1. Introduction

Obesity, defined by the World Health Organization (WHO) as a body mass index (BMI) of ≥30 kg/m^2^ is a rising pandemic. More than 650 million adults were obese worldwide in 2016 [1]. Data from 288 countries involving 13.2 million individuals aged ≥15 years showed global central obesity of 41.5% [2]. The Arab Gulf countries (Saudi Arabia, Qatar, Kuwait, Oman, Bahrain and the United Arab Emirates) have one of the highest prevalence of obesity globally [3,4,5]. According to the WHO, the prevalence rate of obesity in 2016 among adults in Kuwait was 37% followed by Saudi Arabia at 35% [6]. Between 1989 and 2017, the overweight and obesity incidence in the UAE is thought to have more than doubled [5].

According to 2016 WHO statistics, the UAE has a 30% obesity prevalence rate among adults, with a higher incidence among females than males (39% vs. 27%) [6]. Rapid urbanization and economic expansion as a result of revenues from the oil are the major contributors to high obesity prevalence in the Arab Gulf countries [5]. This economic development in the UAE and other Arab Gulf countries has led to a nutrition transition characterized by the consumption of energy-dense foods and sedentary lifestyles [7,8].

Obesity is a major public health issue as it increases the risk of multiple chronic diseases, including type 2 diabetes mellitus, fatty liver and cardiovascular diseases (CVD) [2,9,10]. CVD is one of the leading causes of increased morbidity and mortality in obese individuals [2,11]. It contributes to a significant share of mortality among the Arab Gulf countries. In the United Arab Emirates, CVD contributed to 40% of the mortality due to non-communicable diseases (NCDs) in 2016 [6,12].

Obesity is also a major risk factor for abnormal blood lipid profiles (dyslipidemia) [2,11]. Dyslipidemia is abnormal lipoprotein metabolism, which results in elevated total cholesterol, low-density lipoprotein (LDL-C) cholesterol and triglyceride (TG) concentrations or a decrease in the high-density lipoprotein (HDL-C) cholesterol concentration in the blood [12].

Dyslipidemia alone or in combination with other modifiable risk factors, such as diabetes, hypertension, obesity, low physical activity levels and smoking can increase the risk for CVD morbidity and mortality [13]. Studies conducted in the Arab Gulf countries reported associations between the high prevalence of dyslipidemia and obesity [14,15,16]. In the UAE, the co-exitance of obesity and dyslipidemia has been reported in both children [17] and in adults [18,19].

Physical inactivity and poor diet are considered major modifiable risk factors for dyslipidemia [11,20]. Dietary intake surveys have been employed to statistically attain key dietary habits within a community [21]. Dietary patterns are more indicative of disease risk than the intake of particular nutrients or foods [22].

Healthy dietary patterns are characterized by high intakes of fruits, vegetables, whole grains, low and non-fat dairy and lean proteins [23]. A “western dietary pattern”, which is high in cholesterol, refined sugar, total fat and saturated-fat, has been associated with dyslipidemia [20,24,25]. On the other hand, the Mediterranean Diet (MD) and the Dietary Approaches to Stop Hypertension (DASH) diet are recognized as protective and therapeutic dietary patterns against dyslipidemia [20,26,27]. The adoption of healthy dietary patterns, such as the MD and DASH Diet, plays a key role in achieving blood lipid profile targets [28].

Epidemiological studies have shown adherence to MD as a healthy dietary pattern is associated lower risk of chronic diseases, such as cardiovascular disease [27,29]. Moreover, MD is associated with less overweight and more weight loss [30]. MD is characterized by a high intake of vegetables, fruits, whole grains, olive oil, a moderate consumption of fish and poultry and a low intake of sweets, dairy products and red meat [31]. Although the beneficial effects of MD are well-documented [27,29], two previous studies conducted in the UAE found low adherence to a MD [32,33].

Understanding the dietary intake and other risk factors associated with dyslipidemia in the Arab Gulf countries can assist in the development of policies and preventive programs. Although several studies evaluating dietary intakes to assess obesity, overweight and CVD risks have been performed in the Arab Gulf countries [34,35,36,37], such studies are scarce in the UAE.

One of the few studies on dyslipidemia and associated risk factors conducted in the UAE did not include a dietary intake assessment [18]. Thus, the current study aimed to investigate the associations of dyslipidemia with dietary intake, body weight status and sociodemographic factors in a sample of the adult population in the United Arab Emirates. The data used for the study was collected as part of a larger project examining the role of diets and food systems in the prevention of overweight, obesity and non-communicable chronic diseases in the UAE.

## 2. Materials and Methods

### 2.1. Study Design and Participants

This was a cross-sectional study involving web-based questionnaires. The study aimed to recruit 600 adults from the three main emirates of the UAE (Abu Dhabi, Dubai and Sharjah). Abu Dhabi and Dubai are the major emirates, and the population of Sharjah is more than 50% of the remaining emirates of the UAE. Using a proportional quota sampling method, the Federal Competitiveness and Statistics Authority (FCSA) of the UAE, it was calculated to recruit 270 adults from Abu Dhabi, 230 from Dubai and 100 from Sharjah in order to have a representative sample from each of the three emirates.

The following inclusion criteria were used for the recruitment: (1) adults 18 years and older (males and females); (2) UAE nationals and non-nationals; (3) residing in the following three Emirates: Abu Dhabi, Dubai and Sharjah; and (4) fluent in Arabic language. The recruitment targeted three age groups: 18–30 years, 31–49 years and 50 years and older. Data was collected from July to November 2020.

Online recruitment promotion of the survey was sent through social media (including “WhatsApp™”) and emails to inform the targeted community. An email containing a link to the consent form was sent to those who showed an interest to participate. A link to the questionnaire Google document form was shared with potential participants. In addition, a link to the web-based Food Frequency Questionnaire (FFQ) was emailed to those who agreed to participate. Out of the 720 who responded, 610 provided complete information and were included in the analyses. The study was approved by the United Arab Emirates University Social Sciences Research Ethics Committee (Protocol # ERS_2020_6191). All procedures were following the principles of the Declaration of Helsinki.

### 2.2. Data Collection

Data was collected online using the WHO STEPwise Approach to NCD Risk Factor Surveillance (STEPS) for collecting data on NCDs [38] and a quantitative FFQ.

#### 2.2.1. STEPS Instrument

The STEPS instrument developed by WHO is a cross-cultural validated tool designed to produce results that enables the comparison of data across countries [39]. It was designed for the measurement and surveillance of non-communicable disease risk factors in populations [39]. The STEPS instrument can be used to collect information about risk factors of NCDs, including physical inactivity and raised blood lipids, increased blood glucose and blood pressure. It covers three different levels of “steps” of risk factor assessment (a questionnaire, physical measurements and biochemical measurements). As the study was conducted online during the COVID-19 pandemic, only the questionnaire component of the STEPS Instrument was used (i.e., actual body measurements and biochemical analysis were not performed).

Arabic is the dominant language in the UAE; thus, the Arabic version of the STEPS instrument was adopted in the UAE setting. The original WHO STEPS questions were modified to make them more compatible with the UAE context by removing questions related to alcohol, replacing some of the tobacco products with those commonly used in the UAE and adding additional examples of high sodium processed food items that are commonly used in the UAE. The Arabic version was previously used in the UAE National Nutrition and Health Survey conducted by the Ministry of Health and Prevention in 2017–2018 [40].

The following Core sections of the STEPS Instrument were included in the study: (1) Basic demographic information; (2) Tobacco smoking; (3) Physical activity; (4) History of raised blood pressure; (5) History of raised total cholesterol; (6) History of cardiovascular diseases; and (7) Lifestyle advice. In addition, the expanded section of the STEPS Instrument was used to assess sedentary behavior.

The following questions related to dyslipidemia in the STEPS Instrument were used: (1) Have you ever had your cholesterol (fat levels in your blood) measured by a doctor or other health worker? (2) Have you ever been told by a doctor or other health worker that you have raised cholesterol? (3) Were you first told of this condition in the past 12 months? (4) In the past two weeks, have you taken any oral treatment (medication) for raised total cholesterol prescribed by a doctor or other health worker? (5) Have you ever seen a traditional healer for raised cholesterol? (6) Are you currently taking any herbal or traditional remedy for your raised cholesterol?

The following questions related to the diagnosis of diabetes, hypertension and cardiovascular diseases in the STEPS Instrument were also asked: Have you ever been told by a doctor or other health worker that you have raised blood pressure or hypertension? Have you ever been told by a doctor or other health worker that you have raised blood sugar or diabetes? Have you ever had a heart attack or chest pain from heart disease (angina) or a stroke (cerebrovascular accident or incident)?

Data related to lifestyle advice and dietary modification given to participants by health care professionals in the STEPS instrument were also collected, including advice on quitting smoking, reducing fat intake, increasing intake of fruits and vegetables and maintaining a healthy body weight.

#### 2.2.2. Quantitative Food Frequency Questionnaire

A validated web-based quantitative food frequency questionnaire (FFQ) developed for the UAE adult population [41] was used to assess dietary intake during the month preceding the interview date. Details of the FFQ were previously reported [41]. Briefly, the FFQ is in Arabic and contains 135 food items and 12 food groups that are commonly consumed in the UAE: (1) Dairy foods, (2) Composite dishes, (3) Proteins (including vegetarian and animal sources of proteins), (4) Vegetables (fresh and cooked vegetables including potatoes), (5) Cereals (pasta and other cereals), rice and starches, (6) Sandwiches and baked snacks, (7) Bread and savory biscuits, (8) Spreads on bread, vegetables and salads (excluding use in cooking), (9) Soups, (10) Fruits and dried fruits, (11) Beverages and (12) Sweets and other snacks.

The FFQ contains culturally specific food images depicting the range of distribution of intake among Emirati adults. The food images are presented in a series of three digital food photographs [33]. The food frequency questionnaire contains nine response options: (1) Never or less than once per month; (2) one to two times per month; (3) three times per month; (4) once per week; (5) two times per week; (6) three to four times per week; (7) five to six times per week; (7) Once per day; (8) two times per day; and (9) three times per day.

The daily energy and nutrient intakes of the surveyed participants were calculated from the FFQ. Macronutrients with established recommended dietary intakes were further examined by generating categorical variables based on those values and the percentage of the population above the recommended levels of saturated fat and cholesterol or below for fiber. The database used in the FFQ for the calculation of energy and nutrients intake has been previously reported [42].

### 2.3. Assessment Adherence to Mediterranean Diet

Data from the FFQ was used to examine participants’ adherence to the Mediterranean Diet (MD) using the Composite Mediterranean score (c-MEDS). In the calculation of the c-MEDS, eight food groups used in a previous UAE study to determine adherence to the MD [34] were considered: whole grains, refined grains, legumes, fruits, vegetables, fish, sweetened beverages and olive-oil-to-saturated fat ratio. The c-MEDS was assessed by assigning a score of 1 for consuming equal to or above the median intake of the specific food group and a score of zero for consuming below the median intake [33]. Sugar-sweetened beverages and refined grains were reverse coded.

The sum of the scores of the eight food groups was used to calculate the c-MEDS with a maximum possible score of 8 with higher scores indicating better adherence to the MD. Based on the c-MEDS calculation, participants were categorized either into the “high” or “low adherence” group. Individuals with a c-MEDS below the median score of the population were categorized as a “low adherence” group and those with a c-MEDS equal to or above the median intake of the population were categorized as a “high adherence” group. In addition, intake of eight c-MED food groups of the participants with dyslipidemia and those without were compared.

Food groups were analyzed either as daily or weekly intake. Vegetables, fruit and 100% fruit juice, whole grains and refined grains were calculated as daily intake. Legumes/pulses, fish and sugar-sweetened beverages (SSBs) and the ratio olive-oil-to-saturated-fat were calculated based on the weekly consumption. Composite dishes of the FFQ were broken down into individual ingredients by weight and included in the calculation of the corresponding c-MED food groups. The specific food items considered in each of the food groups are provided as Supplementary Table S1.

### 2.4. Statistical Analysis

Categorical data were presented in frequencies and percentages (*n*, %). Continuous data were screened for extreme outliers and potential misclassifications by tabulation techniques and the use of scatter plots. K density and P-P plots were used to examine distribution. Normally distributed variables were summarized using means and standard deviations (sd), whereas the medians and range (25th–75th percentiles, to avoid extreme variables) were used for skewed data. Student’s *t*-test (for normally distributed) or the Mann–Whitney U test (for skewed data) tests was employed to examine significant differences between continuous variables. The Chi-square test or Fisher’s exact test (for small sub-samples; *n* < 5) was used to examine significant group differences between categorical variables.

Multiple logistic regression was used to assess the association between adherence to the Mediterranean diet and age group, gender, nationality, educational level, employment status, marital status, physical activity level, smoking status, BMI, hypertension diagnosis, diabetes diagnosis and history of cardiovascular disease. Statistical analysis was performed using Stata, version 16.1 [43]; *p*-values < 0.05 were considered to be significant.

## 3. Results

### 3.1. Socio-Demographic Characteristics

Table 1 presents the association of sociodemographic characteristics, BMI, physical activity levels and reported chronic disease diagnosis of the 610 participants in the study. There were 75 participants with and 535 participants without dyslipidemia. Participants with dyslipidemia were significantly older than participants without (41.92 vs. 33.49; *p* < 0.001). There were no significant differences in the gender, nationality and educational level between participants with and those without dyslipidemia.

There was a significant difference in the BMI between participants with and those without dyslipidemia (*p* = 0.002). A total of 72% percent of participants with dyslipidemia were overweight/obese, while 51.2% of participants without were overweight/obese. Moreover, a larger proportion of participants without dyslipidemia were of normal BMI compared to those with dyslipidemia (43.6% vs. 26.7%). Surveyed participants with dyslipidemia reported a significantly higher prevalence of hypertension (40% vs. 10.8%), diabetes (17.3% vs. 4.5%) and history of cardiovascular disease (12% vs. 1.5%) compared to those without dyslipidemia.

### 3.2. Diagnosis and Treatment of Dyslipidemia

Table 2 shows the proportion of the surveyed participants diagnosed with dyslipidemia and treatment. Of the 610 participants, 319 (52.3%) had their blood cholesterol checked in their lifetime, and out of those, 75 (23.5%) reported that they were diagnosed with high blood cholesterol. Of those treated, 38.7% received medical treatment for dyslipidemia, and 20% were taking herbal/traditional remedies.

### 3.3. Energy and Nutrient Intakes

Table 3 shows an intake comparison of energy and macronutrients of the surveyed participants with and without dyslipidemia. There was no significant difference in energy intake between participants with and without dyslipidemia (2202.9 kcal and 2247.8 kcal/day, respectively). However, participants without dyslipidemia obtained a larger proportion of their calories from carbohydrates compared to those with dyslipidemia (50.16% vs. 48.04%, *p* < 0.05).

On the other hand, participants with dyslipidemia obtained a higher proportion of energy from total fat compared to those without dyslipidemia (8.38% vs. 5.79%, *p* = 0.009). There was no significant difference in the proportion of energy from saturated fatty acids between the two groups. However, participants with dyslipidemia reported obtaining a higher proportion of calories from monounsaturated fatty acids (5.16 vs. 2.76, *p* < 0.001) and polyunsaturated fatty acids (5.16 vs. 2.76, *p* < 0.001), compared to those without dyslipidemia.

Over 75% of participants in both groups reported an intake of over 200 g/day of cholesterol. On the other hand, 61.31% of all surveyed participants reported a dietary fiber intake of less than 25 g/day. A comparison of the micronutrient intake between participants with and those without dyslipidemia did not show any significant differences (Supplementary Material—Table S2).

### 3.4. Adherence to Mediterranean Diet

Associations between adherence to the Mediterranean Diet and the sociodemographic characteristics, BMI, physical activity levels and chronic disease diagnosis are presented in Table 4. The median composite MED score (c-MEDS) was 4 with a range between 0 and 8. According to the bivariate (or crude) analysis, age group, nationality, marital status, educational level, smoking status and self-reported diagnosis of dyslipidemia or hypertension were significantly associated with higher adherence to the MD diet.

More specifically, the older the participants were, the higher the likelihood of participants having a higher MD adherence score compared to younger adults (31–49-year old’s: OR: 1.87; 95%CI: 1.26–2.77 and those aged 50+: OR: 4.16; 95%CI: 2.59–6.69 compared to 18–30 years old adults). In the fully adjusted model, only participants aged 50+ years had significantly higher odds of MD adherence score compared to 18–30-year-old participants (OR: 2.97; 95%CI: 1.49–5.92).

In terms of nationality, non-Emirati women were 46% more likely to have high adherence to the MD diet compared to non-Emirati. The likelihood further increased (OR: 1.95; 95%CI: 1.33–2.87) when the model was adjusted for all other factors related to MD. Furthermore, participants who were married or divorced/widowed were also more likely to have a higher MD score compared to single (OR: 2.37; 95%CI: 1.69–3.33 and OR: 2.63; 95%CI: 1.13–6.15, respectively); however, the effect was nulled in the fully adjusted model.

High school graduates and those with post-secondary education were 70% and 67% less likely to have a high MD adherence (OR: 0.30; 95%CI: 0.11–0.79 and OR: 0.33; 95%CI: 0.13–0.83, respectively), compared to those with <12 years of schooling; however, the effect was nulled in the fully adjusted model, and no significant associations remained in any of the categories.

Compared to never smokers, current smokers were 34% less likely to have a high MD adherence score, although this was only seen in the bivariate analysis (OR: 0.64; 95%CI: 0.41–0.98). No effect was found for ex-smokers in any of the models. Lastly, in the bivariate analysis, participants with dyslipidemia or hypertension were also more likely to have a high MD adherence (OR: 1.80; 95%CI: 1.07–3.05 and OR: 2.19; 95%CI: 1.32–3.64, respectively), compared to healthy participants, although the effect was not significant in the adjusted model. Overall, in the fully adjusted model, participants with higher MD scores were non-Emirati and those aged 50+.

The daily and weekly median intakes of C-MED food groups of participants with and those without dyslipidemia are presented in Table 5. There was no significant difference in reported median daily intake of fruits and whole grains between participants with and those without dyslipidemia. Participants with dyslipidemia reported a higher median daily intake of vegetables compared to participants without dyslipidemia (230.28 g vs. 170.03 g; *p* < 0.001). On the other hand, participants without dyslipidemia reported a significantly higher intake of refined grains compared to participants with dyslipidemia (310.85 g vs. 239.96 g, *p* = 0.017).

There was no significant difference in the reported median weekly intake of legumes/pulses, fish and olive-oil-to-saturated-fats between participants with dyslipidemia and those without. Participants without dyslipidemia reported a significantly higher intake of sugar-sweetened beverages compared to those with dyslipidemia (320.95 mL vs. 122.50 mL, respectively; *p* = 0.008).

### 3.5. Advice on Lifestyle and Diet Modifications

**Figure 1:**
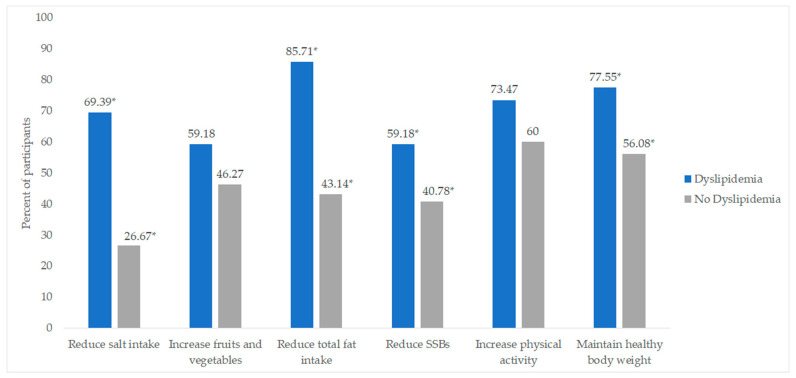
Lifestyle advice and dietary modifications given to the participants by health care professionals. * Significance at a = 5%; comparison between groups performed using the Chi-square test or Fisher’s exact test (for small sub-samples; *n* < 5). SSBs—Sugar-sweetened beverages.

Figure 1 shows the proportions of the participants with and those without dyslipidemia who reported receiving lifestyle and dietary modification advice from doctors and other health professionals. A significantly higher proportion of participants with dyslipidemia received advice related to reducing salt (*p* < 0.001), reducing total fat (*p* < 0.001), reducing sugar-sweetened beverages SSBs (*p* = 0.017) and maintaining a healthy weight (*p* = 0.005).

## 4. Discussion

Dyslipidemia is a major risk factor for cardiovascular disease [44]. A dyslipidemia prevalence of 72.5% among the adult Emirati adults with a mean age of 42.8 years was reported [18], which is greater than the global incidence of 39% [1]. Diet and obesity are modifiable risk factors for dyslipidemia. Due to the limited studies on dietary patterns and dyslipidemia in the UAE, we examined the associations of dyslipidemia with dietary intakes, body weight status and sociodemographic factors in a sample of adults in the United Arab Emirates.

The prevalence of dyslipidemia among participants in the present study who reported that they had their blood cholesterol measured in their lifetime (*n* = 319) was 23.5%, which is much lower than a prevalence of 72.5% reported in a previous UAE study [18]. Possible reasons for this difference include the younger age of participants in the present compared to the previous survey (34.5 vs. 42.8 years, respectively) [18]. In addition, the present study relied on a self-reported diagnosis of dyslipidemia rather than the biochemical assessments as in the previous study [18].

Gender, smoking, age, central obesity and diabetes were the significant predictors of dyslipidemia in the previous study [18], while in the present study, gender was not associated with dyslipidemia. Another study conducted in the UAE involving 5126 young adults, aged 18–40 years found a higher prevalence of dyslipidemia among men compared to women [19]. However, in the above-mentioned two studies, there was a higher proportion of males (51.8% and 62%, respectively) compared to the current study, which involved predominately female participants (64.3%).

Mezhal and colleagues [19] also reported the coexistence of obesity and dyslipidemia with other cardiometabolic risk factors, such as hypertension and diabetes. In the present study, reported dyslipidemia diagnosis was associated with increased age, higher BMI and a history of diabetes, hypertension and cardiovascular disease. The link between obesity and dyslipidemia is well-established [11,44].

Obesity, especially abdominal obesity, is associated with dyslipidemia [45,46]. Among Chinese adults, older age, overweight and obesity were associated with dyslipidemia [45]. Several studies conducted in Saudi Arabia found an association between dyslipidemia with obesity and sociodemographic factors, including high waist circumference, higher BMI and age, within both genders [36] as well as increasing age, male gender and BMI [47].

The United Arab Emirates has undergone major economic changes and urbanization over the past five decades, which is accompanied by nutritional transition changes from the traditional diet to diets higher in fat, sugar and meats and lower in whole grains, fruits and vegetables [7]. This eating pattern is reflected in the results obtained from the dietary intakes of the participants in the current study. For example, over 80% of participants with dyslipidemia and those without had a saturated fat intake greater than 10% of their daily total calories, over 70% had dietary cholesterol greater than 200 mg/day, and 61% of them did not meet the dietary fiber recommendation of at least 25 g/day [48].

An analysis of the consumption of food groups in the composite Mediterranean Diet in the present study showed a higher consumption of vegetables among participants previously diagnosed with dyslipidemia. This may be as a result of the advice of increasing vegetable intake received after the diagnosis of dyslipidemia. On the other hand, participants without a diagnosis of dyslipidemia reported consumption of refined grains and sugar-sweetened beverages. It is possible that participants with dyslipidemia adapted healthier dietary habits after the diagnosis by reducing the intake of refined grains and sugar-sweetened beverages.

In a study by Enani and colleagues, carbonated beverages were associated with an elevated risk of dyslipidemia in males [36]. Among Kuwaiti adults, the risk of metabolic syndrome or dyslipidemia was higher in individuals who adhered more closely to the poultry/refined grains diet pattern than in those who adhered the least [37]. On the other hand, the consumption of a diet high in vegetables was linked to a reduced incidence of dyslipidemia but not the CVD risk variables [37].

Research has confirmed the multiple benefits of healthy dietary patterns, including the Mediterranean diet (MD) and the Dietary Approaches to Stop Hypertension (DASH) Diet, on their protective role against CVD [26,49,50,51,52], and these should be recommended for dyslipidemia prevention and management [44]. In addition to the health benefits, healthy dietary patterns, such as the MD and DASH diet, are higher in plant-based foods and lower in animal-based foods supporting environmental sustainability [28,53]. An analysis of data from a representative sample of Emirati adult women showed that the MD supports environmental sustainability [33].

The current study examined the sociodemographic factors associated with adherence to the MD among the participants. After adjusting for covariates, such as age, gender, nationality, educational level, physical activity level, smoking status, BMI, self-reported diagnosis of hypertension, diabetes and history of cardiovascular disease, the adherence to MD was low (Mediterranean Diet composite Score of 4 out of 8), Our findings are consistent with the results of a previous study involving adult Emirati women aged 19–50 years [33] as well as study conducted in UAE during the COVID_19 lockdown [32]. Moreover, the results showed higher adherence to the MD among older participants.

Similarly, a study conducted in Kuwait found that older adults had higher adherence to the vegetable-rich dietary pattern while younger adults had higher adherence to the fast-food or refined-grains/poultry dietary patterns [37]. A study conducted in Southern Italy and Malta also showed higher adherence to MD among older participants [54,55]. We also found that non-Emirati participants had higher adherence to the MD compared to the UAE nationals. This might be because most of the non-Emirati participants were originally from countries in the Middle East, such as Lebanon, Jordan and Syria, that traditionally follow the MD.

The findings of the current study highlight the need to develop intervention programs promoting the adoption of dietary choices, which are consistent with cardiovascular health-promoting dietary patterns, such as the MD [29,50] and the DASH diet [26,52]. These programs should specifically target younger adults, since based on the present study they were less adherent to MD compared to those who were older. In a recent study involving university students in the UAE, only 28.7% and 34% reported eating fruits and vegetables daily, respectively [56].

Educational initiatives targeting the prevention of obesity through public awareness and other initiatives are needed to mitigate dyslipidemia and associated CVD in the UAE. Moreover, screening for dyslipidemia in young adults could be considered a promising strategy for reduction in risk factors and CVDs. A recent study conducted in the found a high prevalence of cardiometabolic risk factors among adults aged 18–40 years [19].

The results from the present study found that 20% of those diagnosed with high cholesterol reported taking herbal/traditional remedies. In a previous study conducted in the UAE, 39.3% of participants with diabetes reported using complementary and alternative medicine (CAM) since diagnosis [57]. Some of the CAM used by the surveyed participants included herbs, vitamins and mineral supplements [57]. Therefore, there is a need to increase awareness of the safety of CAM among patients with chronic diseases.

There is a need for public awareness and policies that facilitate the consumption of health-promoting dietary patterns. Greater efforts should be placed to access of affordable healthy foods, such as fruits, vegetables and whole grains. Higher food prices, such as fruits and vegetables were identified to be a barrier to adherence to the MD in other countries [58,59].

A range of interventions at different levels, including accessible and affordable healthy foods, have a more promising approach to reducing the prevalence of obesity and other risk factors of dyslipidemia and CVD. These interventions should combine individual-level behavioral interventions with changes in the food environment in and the cultural context of the UAE society. Moreover, early identification and management of dyslipidemia and other risk factors of CVD at an earlier age are needed. In the present study, only 52.3% of the participants had their blood cholesterol measured in their lifetime.

There are several strengths of this study. To the best of our knowledge, this is the first study investigating the dietary patterns of adults in the UAE diagnosed with dyslipidemia using a country-specific food frequency questionnaire. Furthermore, this study included both Emirati nationals and expatriate residents of the UAE. Finally, the use of the WHO STEPS Instrument, which is a valid tool for collecting population-level data on risk factors of CVD [31] is another strength of the study. However, there are several limitations of this study. The cross-sectional nature of the study limits the causality. Another potential limitation is a recall bias related to the self-reported dietary intake and the diagnosis related to dyslipidemia and other chronic diseases.

Moreover, since the survey was conducted online due to the COVID-19 pandemic, actual body measurements and biochemical measurements were not collected. Future studies that include the other components of the STEPS Instruments, such as physical measurements (blood pressure, weight, height and waist circumference) and biochemical measurements (blood glucose and blood lipid profile) are needed. Finally, data used for this study involved only the three main emirates of the UAE; thus, national-level data from a representative sample of the UAE population is needed.

## 5. Conclusions

In this study, we evaluated the diet and other risk factors associated with dyslipidemia in a sample of adults from the UAE, which has a high prevalence of CVD. The results showed that the food choices of adults in the UAE who were with and without dyslipidemia were not consistent with health-promoting dietary patterns, such as the MD. Interventions that combine individual-level behavioral changes with adjustments in the food environment and within the cultural context of the UAE society are needed.

## Figures and Tables

**Table 1:** Association of dyslipidemia with sociodemographic characteristics, BMI, physical activity and chronic diseases.

Variable	Total Population (*n* = 610)	Dyslipidemia * (*n* = 75)	Non-Dyslipidemia (*n* = 535)	*p*-Value ^§^
Age, mean (sd)	34.5 (14.3)	41.92 (14.5)	33.49 (14.0)	<0.001
Age group, *n* (%)				
18–30 years	327 (53.6%)	23 (30.7%)	304 (56.8%)	<0.001
31–49 years	151 (24.8%)	25 (33.3%)	126 (23.6%)	
50+ years	132 (21.6%)	27 (36.0%)	105 (19.6%)	
Gender, *n* (%)				
Females	392 (64.3%)	43 (57.3%)	349 (65.2%)	0.181
Males	218 (35.7%)	32(42.67%)	186 (34.8%)	
Nationality, *n* (%)				
Emirati	202 (33.0%)	22 (29.3%)	180 (33.6%)	0.470
Expat	408 (67.0%)	53 (70.7%)	355 (66.4%)	
Marital Status, *n* (%)				
Single	312 (51.1%)	24 (32.0%)	288 (53.8%)	<0.001
Married	270 (44.3%)	47 (62.7%)	223 (41.7%)	
Divorced + Widowed	28 (4.6%)	4 (5.3%)	24 (4.5%)	
Educational level, *n* (%)				
<12 years	31 (5.1%)	3 (4.0%)	28 (5.2%)	0.363
High school graduate	117 (19.2%)	10 (13.3%)	107 (20.0%)	
Post-Secondary	462 (75.7%)	62 (82.7%)	400 (74.8%)	
Smoking status, *n* (%)				
Current	101 (16.6%)	17 (22.7%)	84 (15.7%)	0.292
Never	473 (77.5%)	55 (73.3%)	418 (78.1%)	
Ex-smoker	36 (5.9%)	3 (4.0%)	33 (6.2%)	
BMI, *n* (%)				
Underweight	29 (4.8%)	1 (1.3%)	28 (5.23%)	0.002
Normal	253 (41.5%)	20 (26.7%)	233 (43.6%)	
Overweight/Obese	328 (53.8%)	54 (72.0%)	274 (51.2%)	
Physical activity level, *n* (%)				
Sedentary	183 (30.0%)	17 (22.7%)	166 (31.0%)	0.182
Low	91 (14.9%)	14 (18.7%)	77 (14.4%)	
Moderate	195 (32.0%)	21 (28.0%)	174 (32.5%)	
High	141 (23.1%)	23 (30.7%)	118 (22.1%)	
Hypertension, *n* (%) *	88 (14.4%)	30 (40.0%)	58 (10.8%)	<0.001
Diabetes, *n* (%) *	37 (6.1%)	13 (17.3%)	24 (4.5%)	<0.001
Cardiovascular disease, *n* (%) *	17 (2.8%)	9 (12.0%)	8 (1.5%)	<0.001

**^§^** Significance at a = 5%; comparison between groups performed using chi-square test or Fisher’s exact test (for small sub samples; *n* < 5), comparison between continuous variables were performed using an independent *t*-test. * self-reported based on a previous diagnosis by a doctor or another healthcare professional.

**Table 2:** Diagnosis and treatment of dyslipidemia by gender.

Variable	Total Population	Female	Male	*p*-Value ^§^
Have you ever had your cholesterol (fat levels in your blood) measured by a doctor or other health worker? (*n* = 610)	319 (52.3%)	209 (53.3%)	110 (50.5%)	0.498
Diagnosed with raised blood cholesterol (*n* = 319)	75 (23.5%)	43 (11.0%)	32 (14.7%)	0.181
Diagnosed with raised blood cholesterol in the past year (*n* = 75)	47 (62.7%)	25 (58.1%)	22 (68.8%)	0.347
Took medication for cholesterol during the past 2 weeks (*n* = 75)	29 (38.7%)	15 (34.9%)	14 (43.8%)	0.435
Saw a traditional healer for cholesterol (*n* = 75)	21 (28.0%)	11 (25.6%)	10 (31.3%)	0.589
Currently taking herbal/traditional remedy for cholesterol (*n* = 75)	15 (20.0%)	10 (23.3%)	5 (15.6%)	0.414

^§^ Significance at a = 5%; comparison between groups assessed using chi square test or Fischer exact test for cells with *n* ≤ 5.

**Table 3:** Macronutrient intakes of adults with and without dyslipidemia.

Variable	Total Population (*n* = 610)	Dyslipidemia (*n* = 75)	Non-Dyslipidemia (*n* = 535)	*p*-Value ^§^
Energy (kcal/day), mean (sd)	2242.30 (890.61)	2202.87 (872.95)	2247.82 (893.72)	0.681
Protein (g/day)	91.26 (41.12)	89.67 (39.82)	91.48 (41.33)	0.722
Protein (g/kg/day)	1.34 (0.64)	1.23 (0.61)	1.36 (0.64)	0.115
Protein (% energy)	16.33 (3.42)	16.35 (3.93)	16.32 (3.35)	0.943
Carbohydrates (% energy)	49.90 (8.11)	48.04 (9.34)	50.16 (7.90)	0.034
Total sugars (% energy)	17.64 (6.80)	17.28 (7.91)	17.69 (6.64)	0.622
Total Fat (% energy)	35.12 (6.19)	37.34 (8.38)	34.81 (5.76)	0.009
Saturated fat (sfa, % energy)	12.12 (2.99)	12.49 (3.25)	12.07 (2.95)	0.253
Percent participants with saturated fat acids > 10%, *n* (%)	495 (81.15%)	64 (85.33%)	431 (80.56%)	0.322
Polyunsaturated fat (% energy)	13.39 (3.20)	14.93 (5.16)	13.17 (2.76)	<0.001
Monounsaturated fat (% energy)	13.38 (3.20)	14.93 (5.16)	13.16 (2.76)	<0.001
Cholesterol (g/day), median (25–75%)	319.49 (208.86, 447.45)	361.97 (219.1, 479.07)	311.15 205.35, 444.4)	0.175
Percent participants with dietary cholesterol > 200 g, *n* (%)	463 (75.90%)	57 (76.00%)	406 (75.89%)	0.983
Fiber (g/day), median (25–75%)	22.29 (15.92, 30.51)	21.67 (16.76, 29.57)	22.45 (15.92, 30.60)	0.909
Percent participants with dietary fiber < 25 g, *n* (%)	374 (61.31%)	46 (61.33%)	328 (61.31%)	0.997

^§^ Significance at a = 5%; Comparisons between continuous variables with normal distribution were performed using an independent *t*-test, comparison between skewed variables were performed using the Mann–Whitney U test, and comparisons between categorical were performed using the Chi-square test.

**Table 4:** Characteristics associated with adherence to the Mediterranean Diet among the surveyed participants.

Variable	Total Population (*n* = 610)	Adherence to MD ^‡^ 4 (3–5)		OR 95% CI ^¥^	OR 95% CI ^§^
		Low (*n* = 251)	High (*n* = 359)		
Age					
18–30 years ^Ω^	327 (53.6%)	169 (67.3%)	158 (44.0%)	-	-
31–49 years	151 (24.8%)	55 (21.9%)	96 (26.7%)	1.87 * (1.26–2.77)	1.53 (0.86–2.69)
50+ years	132 (21.6%)	27 (10.8%)	105 (29.3%)	4.16 * (2.59–6.69)	2.97 * (1.49–5.92)
Gender					
Female ^Ω^	392 (64.3%)	166 (66.1%)	226 (63.0%)	-	-
Male	218 (35.7%)	85 (33.9%)	133 (37.1%)	1.15 (0.82–1.61)	0.93 (0.63–1.38)
Nationality					
Emirati ^Ω^	202 (33.0%)	95 (38.0%)	106 (29.5%)	-	-
Non-Emirati	408 (67.0%)	155 (62.0%)	253 (70.5%)	1.46 * (1.04–2.06)	1.95 * (1.33–2.87)
Marital Status					
Single ^Ω^	312 (51.2%)	160 (63.8%)	152 (42.3%)	-	-
Married	270 (44.3%)	83 (33.1%)	187 (52.1%)	2.37 * (1.69–3.33)	1.43 (0.84–2.45)
Divorced/ Widowed	28 (4.6%)	8 (3.2%)	20 (5.6%)	2.63 * (1.13–6.15)	1.33 (0.48–3.70)
Educational level					
<12 years ^Ω^	31 (5.1%)	6 (2.4%)	25 (7.0%)	-	-
High school Graduate	117 (19.2%)	52 (20.7%)	65 (18.1%)	0.30 * (0.11–0.79)	0.39 (0.14–1.11)
Post-Secondary	462 (75.7%)	193 (76.9%)	269 (74.9%)	0.33 * (0.13–0.83)	0.41 (0.15–1.11)
Smoking status					
Current	101 (16.6%)	51 (20.3%)	50 (13.9%)	0.64 * (0.41–0.98)	1.71 (1.05–2.77)
Never ^Ω^	473 (77.5%)	186 (74.1%)	287 (79.9%)	-	-
Ex-smoker	36 (5.9%)	14 (5.6%)	22 (6.1%)	1.02 (0.51–2.04)	1.60 (0.70–3.65)
BMI					
Underweight	29 (4.8%)	18 (7.2%)	11 (3.1%)	0.49 (0.22–0.07)	0.56 (0.25–1.25)
Normal ^Ω^	253 (41.5%)	112 (44.6%)	141 (39.3%)	-	-
Overweight/Obese	328 (53.8%)	121 (48.2%)	207 (57.7%)	1.36 (0.97–1.90)	0.91 (0.62–1.36)
Physical Activity level					
Sedentary ^Ω^	183 (30.0%)	79 (31.5%)	104 (29.0%)	-	-
Low	91 (14.9%)	39 (15.5%)	52 (14.5%)	1.09 (0.65–1.82)	0.10 (0.57–1.73)
Moderate	195 (32.0%)	76 (30.3%)	119 (33.2%)	1.19 (0.78–1.80)	1.16 (0.75–1.81)
High	141 (23.1%)	57 (22.7%)	84 (23.4%)	1.13 (0.72–1.77)	1.38 (0.85–2.24)
Dyslipidemia	75 (12.3%)	22 (8.8%)	53 (14.8%)	1.80 * (1.07–3.05)	1.33 (0.72–2.39)
Hypertension	88 (14.4%)	23 (9.2%)	65 (18.1%)	2.19 * (1.32–3.64)	1.71 (0.961–3.02)
Diabetes	37 (6.1%)	16 (6.4%)	21 (5.9%)	0.91 (0.47–1.79)	0.55 (0.26–1.17)
Cardiovascular disease	17 (2.8%)	5 (2.0%)	12 (3.3%)	1.70 (0.59–4.89)	0.99 (0.30–3.26)

^¥^ Significance at a = 5%; bivariate analysis performed using logistic regression with adherence to MD as the dependent variable. ^§^ Significance at a = 5%; analysis performed using multiple logistic regression with adherence to MD as the dependent variable and age group, gender, nationality, educational level, employment status, marital status, physical activity level, smoking status, BMI, hypertension diagnosis, diabetes diagnosis and history of cardiovascular disease as independent variables. ^‡^ median (25–75%); Low MD adherence: c-MEDS < median score; High MD adherence: c-MEDS ≥ median score. * *p* < 0.05. MD-Mediterranean Diet. c-MEDS-Composite Mediterranean Diet Score. ^Ω^ Reference category. All variables presented as *n* (%).

**Table 5:** c-MED Food group intakes of adults with and without dyslipidemia.

Variable	Total Population (*n* = 610)	Dyslipidemia (*n* = 75)	Non-Dyslipidemia (*n* = 535)	*p*-Value ^§^
Daily intake				
Vegetables (g)	175.43 (89.23–297.91)	230.28 (144.29–402.52)	170.03 (85.93–282.92)	0.004
Fruit & 100% fruit juice (g)	177.72 (84.12–319.84)	193.89 (79.25–360.74)	175.91 (84.12–316.75)	0.397
Whole grains (g)	33.61 (14.32–62.98)	31.39 (13.39–53.37)	33.77 (14.65–63.72)	0.662
Refined grains (g)	293.12 (187.36–452.75)	239.96 (133.44–407.65)	310.85 (190.31–456.91)	0.017
Weekly intake				
Legumes/pulses (g)	125.40 (69.58–243.50)	110.25 (60.00–221.7)	129.00 (70.04–256.54)	0.325
Fish (g)	174.47 (47.04–340.97)	199.19 (63.70–396.32)	173.95 (47.04–340.75)	0.264
Sugar-sweetened beverages (mL)	300.00 (73.50–855.50)	122.50 (0.00–710.00)	320.95 (73.50–899.50)	0.008
Olive oil to Saturated fats ratio	0.917 (0–1.5)	0.900 (0–1.241)	0.917 (0–1.5)	0.457

^§^ Comparison between groups performed using the Mann–Whitney U test. All values are presented as median (25–75%). c-MED—Composite Mediterranean Diet.

## Data Availability

The dataset used to prepare this analysis is available from the corresponding authors.

## Supplementary Materials

The following supporting information can be downloaded at: https://www.mdpi.com/article/10.3390/nu14163405/s1, Table S1: Micronutrient intakes in adults with and without dyslipidemia; Table S2: c-MED food group items.
