# Supplementary material for: Associations of Dyslipidemia with Dietary Intakes, Body Weight Status and Sociodemographic Factors among Adults in the United Arab Emirates
Source: Nutrients. 2022 Aug 19;14(16):3405. doi: 10.3390/nu14163405 (PMC9412376; doi:10.3390/nu14163405)
Supplement: Supplementary file 1 [file nutrients-14-03405-s001.zip › Table S1 Micronutrient intakes.pdf]

Table S1: Micronutrient intakes in adults with and without dyslipidemia

Variables	Total population (n=610)	Dyslipidemia (n=75)	Non-Dyslipidemia (n=535)	P-value§
Vitamin C	155.2 (100.4, 231.1)	158.0 (109.2,237.0)	155.16 (99.0,230.5)	0.879
Vitamin D	4.5 (2.7, 7.1)	3.8 (2.5,7.1)	4.6 (2.8,7.2)	0.160
Vitamin E	8.5 (6.0, 11.9)	9.2 (6.1, 13.2)	8.4 (5.9,11.9)	0.135
Thiamin	2.4 (1.5, 3.7)	2.3 (1.3,3.7)	2.4 (1.5,3.7)	0.310
Riboflavin	2.4 (1.5, 4.7)	2.2 (1.4, 4.0)	2.4 (1.5, 4.2)	0.243
Pyridoxine	2.5 (1.8, 3.4)	2.4 (1.8,3.5)	2.5 (1.8,3.4)	0.976
Folate	327.3 (237.8, 467.4)	339.2 (248.8,488.8)	325.4 (236.1, 464.8)	0.484
vitamin B_{12}	6.0 (3.4, 9.9)	7.02 (3.3,11.0)	5.8 (3.4, 9.8)	0.523
Calcium (mg)	787.6 (541.4, 1061.6)	711.8 (464.2,1076.8)	800.9 (544.4,1059.9)	0.214
Iron (mg)	14.0 (10.1, 19.0)	13.2 (9.7,19.3)	14.2 (10.1,19.0)	0.495

§Significance at a=5%; Comparisons between variables were performed using Kruskal Wallis test. All values presented as median (25% -75%)
