# Supplementary material for: Associations of Dyslipidemia with Dietary Intakes, Body Weight Status and Sociodemographic Factors among Adults in the United Arab Emirates
Source: Nutrients. 2022 Aug 19;14(16):3405. doi: 10.3390/nu14163405 (PMC9412376; doi:10.3390/nu14163405)
Supplement: Supplementary file 1 [file nutrients-14-03405-s001.zip › Table S2 - c-MED Food group item.pdf]

Table S2: c-MED food group items

Food Group	Food Items
Vegetables	All salads and vegetables, including starchy vegetables (except French fries) and calculated portions from mixed dishes containing vegetables and vegetable-based soups (e.g. stuffed grape leaves and cabbage, tabbouleh, oat/vegetable soup)
Fruit & 100% fruit juice	All fresh and dried fruits and 100% fruit juices
Whole grains	All whole wheat bread and cereals
Refined grains	All white types of breads, cereal, rice, pasta, and calculated portions from mixed dishes containing refined grain products
Legumes/pulses	Baked beans, red beans, broad beans, lentils, chickpeas and calculated portions from mixed dishes containing legumes/ (e.g. hummus & falafel)
Fish	All fish and seafood, calculated fish portions from mixed dishes containing fish
Sugar-sweetened beverages	All drinks containing sugar (e.g. soft drinks, sweetened fruit drinks, sweetened tea and coffee)
Olive oil - Saturated fat ratio	Includes olive oil, olives, butter, ghee

c-MED – Composite Mediterranean Diet
